# From grassroots to global: A blueprint for building a reproducibility network

**DOI:** 10.1371/journal.pbio.3001461

**Published:** 2021-11-10

**Authors:** UK Reproducibility Network Steering Committee

**Affiliations:** School of Psychological Science, University of Bristol, Bristol, United Kingdom

## Abstract

Researchers, institutions, funders, and publishers are considering how to improve research culture and quality, but no single part of the research ecosystem can effect change on its own. The UK Reproducibility Network (UKRN) was established to facilitate the necessary coordination. Its experience can inform the establishment of like-minded networks around the world to drive positive change.

Change is in the air. In the United Kingdom, we have seen the publication of a major report on research incentives, commissioned by UK Research and Innovation [1], while Wellcome has initiated a programme of activity around research culture, and the UK Government has launched its R&D People and Culture Strategy [2]. This is motivated, in part, by the realisation that many of the working practices of academic research remain rooted in the 19th century model of the independent researcher. But research has changed—there is a far greater need to work in multidisciplinary teams, and the tools available to us are unrecognisable from those available even 10 years ago. The scope to make, not only our research outputs, but our entire research workflows, openly available for scrutiny and reuse is far greater now, and we can use this to recognise the many and granular contributions of individual researchers to a project.

This change is happening in many places. Individual researchers are adopting open research practices (and using the growing number of tools and platforms that support this). Funders are mandating data sharing and open access publishing, recognising preprints, and developing new CV formats. Publishers are similarly encouraging transparency, for example, through the Transparency and Openness Promotion Guidelines [3]. Moreover, we are seeing innovative partnerships, such as Registered Reports Funding Partnerships between funders and journals, which aim to streamline the process of applying for funding and submitting a Registered Report, in the hope that this will improve both efficiency and quality [4].

But this activity risks being inefficient. Multiple solutions to the same underlying problem may be generated, and gaps may remain, if this change is allowed to develop entirely organically. There is certainly space for multiple potential solutions, so that innovation can flourish (adaptive radiation!). But there is also a need for some degree of coordination and harmonisation. For example, if an institution begins to incentivise open research practices, by including these in promotion criteria, researchers at that institution may be at a disadvantage if they subsequently move to a different institution where these practices are not rewarded. We are facing a collective action problem and therefore need to act collectively where possible.

This was the motivation behind the establishment of the UK Reproducibility Network (UKRN; www.ukrn.org) in 2019, which has led to the emergence of similarly structured national reproducibility networks in other countries (see Fig 1). These are all modelled along similar lines, intended to connect the grassroots community of researchers with institutions, as well as funders, publishers, learned societies, and other sectoral organisations. Each network has 3 main interacting elements: local networks (informal, self-organising groups of researchers and other staff at individual institutions, represented by a Local Network Lead); institutions (universities that have formally joined the Network by creating a senior academic role focused on research improvement); and other sectoral organisations (funders, publishers, learned societies and so on, which all have a stake in the quality of research).

**Fig 1:**
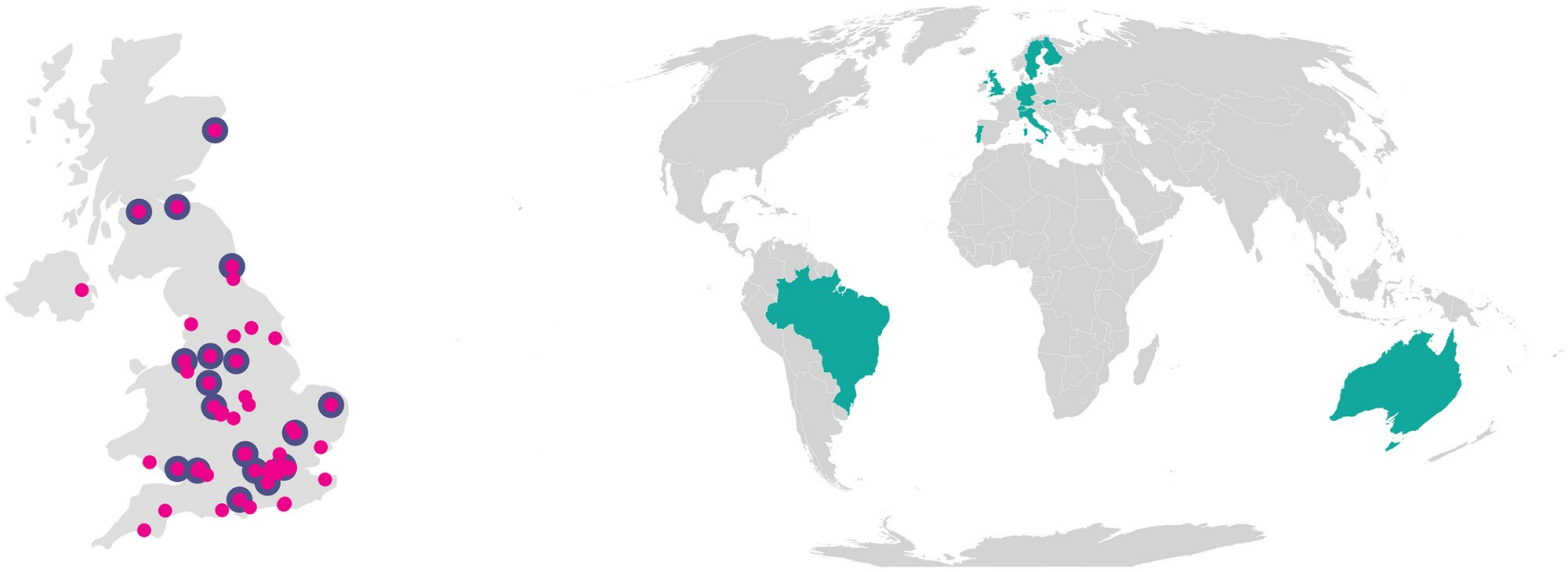
The UKRN and beyond. Left panel: As of October 25, 2021, the UKRN comprised 57 local networks (https://www.ukrn.org/local-network-leads), shown in pink on the UK figure, and 21 institutional members (https://www.ukrn.org/institutional-leads), shown in purple. We also have 37 members of our external stakeholder group, comprising funders, publishers, learned societies, and other sectoral organisations (https://www.ukrn.org/stakeholders). As UKRN grows, these numbers change! Right panel: UKRN is only one of a growing number of national reproducibility networks worldwide (https://www.ukrn.org/international-networks), in Europe and beyond. *Image credit*: *Adam Kenny*. The base layer of the figure is from the package “maps” in R (https://CRAN.R-project.org/package=maps). UKRN, UK Reproducibility Network.

Reproducibility networks are fundamentally peer-led organisations, with the aim of raising research quality and promoting initiatives that may help achieve this, as well as supporting a positive research culture. This includes the investigation of factors that contribute to robust research, promoting training activities and disseminating best practice, and working across local networks, institutions, and external stakeholders to ensure coordination of efforts across the sector. The key feature of reproducibility networks is their structure, which is flexible enough to allow for national, institutional, and disciplinary differences, while also enabling coordination of activity within and between these agents in the research ecosystem. Our approach to setting up UKRN—the first reproducibility network—is outlined in Box 1.

**Box 1. Building a network—The UKRN “origin story”**

The UKRN can be traced back to a meeting held by the Academy of Medical Sciences, jointly with the Biotechnology and Biological Sciences Research Council (BBSRC), Medical Research Council (MRC), and Wellcome in 2015, on the challenges and opportunities for improving the reproducibility and reliability of biomedical research in the UK [5]. At this meeting, it was clear that there was a desire to address these issues, but no single organisation who had clear responsibility for doing so.

A number of academic colleagues began discussing how best to achieve this and lobbied funders for support. In September 2018, we were able to bring several funders, publishers, and other sectoral organisations together at a meeting at the University of Bristol, to discuss a model for coordinating this activity. There was enthusiastic support, and the UKRN was born, launched formally at an event at King’s College London in March 2019.

There was already a large community of researchers engaged with these issues, many of whom volunteered to form local networks at their institutions. This part—the foundation of UKRN—grew rapidly. Our target was to recruit 10 institutions as institutional members in 3 years, but we recruited this number in less than a year [6], driven perhaps by the Research Excellence Framework in the UK, which provided an incentive for institutions to join.

Our model has been to remain light touch and flexible—our Terms of Reference are brief, and while they include model role descriptions for Local Network Leads and Institutional Leads, these are intended to be a starting point, rather than prescriptive. We recognise that while there is value in coordinating activity, each discipline, institution, and country (as we see other national reproducibility networks emerge) will have different specific needs.

We also are able to operate on a modest budget that supports an administrator, our website, and support for key initiatives. Our strategy was to ask for modest support (ranging from £500 to £10,000 per year over 3 years) from a relatively large number of funders. This approach may work best in countries with a sufficient number of funders to support this approach, but it lowered the barrier to entry for these funders, given that the amount requested was modest.

Our medium-term strategy was to use this funding to establish UKRN, and grow the Network, while seeking more substantial funding by the end of that initial 3-year period. In July 2021, we were awarded £4.5 million by Research England (with an additional £4 million in-kind contribution from partners) to continue our activities for a further 5 years, focused on embedding open research practices across our partner institutions and, ultimately, the wider sector [7].

This funding—including the substantial in-kind contributions from our partner institutions and other organisations—provides a stable platform for UKRN for the next 5 years. In that time, we will have to develop a longer-term sustainability model that will allow us to continue our activity. What this looks like remains to be seen, but it is exciting to be able to continue to work collaboratively across the sector, nationally and internationally.

**Lessons learned:**
1. **Offer solutions.** We identified a need for coordination, to galvanise the community of researchers already engaged with issues of reproducibility, but also to provide a mechanism to engage with institutions, funders, publishers, and other organisations—all of whom saw a need to address these issues, but none of whom regarded it as their sole responsibility to coordinate.
2. **Be pragmatic and flexible.** Our initial focus was on growing our community—local networks, institutions, and sectoral organisations. To that end, we deliberately adopted a light touch approach to reduce barriers to entry. While we had some guiding principle—laid out in our Terms of Reference—we understood the need to be flexible given the wide variety of institutions and organisations we were engaging with.
3. **Manage growth.** One of the biggest challenges was the speed with which UKRN grew. Local networks emerged very rapidly, and institutions and other organisations joined much more quickly than anticipated. The distributed nature of UKRN helped us to manage this, but we are acutely aware of the need to support our local networks, in particular, and to ensure the contribution of Local Networks Leads is recognised.

This structure allows us to support activity at the grassroots through our local networks, while also harmonising activity across institutions. For example, in the UK, many of our institutions have introduced Open Research Prizes [8] and are incorporating open research practices into their promotion criteria. It also allows us to broker partnerships between other organisations, such as Registered Reports Funding Partnerships [9,10]. Moreover, it enables coordination across these elements. For example, in the UK, we have been able to link our Local Network Leads directly with major sectoral organisations such as UK Research and Innovation—responsible for directing public research and innovation funding within the UK—when they were consulting on the scope of the planned UK Committee on Research Integrity [11]. We were able to connect our Local Network Leads with key individuals within UKRI, to provide their perspective.

There is considerable diversity across and within these networks—a range of countries with very different national systems and research cultures, and from small to large institutions that also differ along a range of other dimensions. This also allows us to introduce a range of perspectives—in particular across disciplines—so that researchers in different fields can learn from one another. The specific solutions and activities can be local, but the coordination and mutual support are national and, increasingly, international. For example, we have developed a survey of open research practices that we will deploy across reproducibility networks, with a view to benchmarking the current state of open research practices within and across countries. This will allow us to evaluate the impact of our activity over time and monitor change.

The growth of these networks in less than 3 years is extremely encouraging and testament to the energy and enthusiasm of grass roots researchers in particular (and an indication of the wider desire across the sector—from institutions to funders and publishers—to engage and work collaboratively to drive positive change). We have been able to support several grassroots initiatives (https://www.ukrn.org/initiatives), including many developed by early careers researchers, such as the ReproducibiliTea journal club format (https://reproducibilitea.org) and the RIOT Science Club seminar series format (http://riotscience.co.uk). Moreover, we have increasingly become an important source of information for sectoral organisations. Our ability to link researchers with these organisations is key to this.

Ultimately, we will need to demonstrate the value of our work to institutions, funders, and others. In principle, the collaborative approach at the heart of our activity should offer advantages in terms of effectiveness and efficiency—for example, we are developing open research training delivered via train the trainer courses that allow institutions to deliver local workshops while ensuring that the content is coordinated in a way that should serve to improve interoperability and support researcher mobility. This is supported by a growing body of online materials (https://www.ukrn.org/primers/). This approach is also likely to be highly cost-effective, but we will need to evaluate the impact of this approach, both in terms of uptake and in terms of whether it ultimately has a positive effect on research quality.

The research ecosystem is—of course—global, not national. It is therefore particularly exciting to see the rapid emergence of other national reproducibility networks, modelled on the UKRN. Critically, the coordination that these provide should be informed by the voices of researchers themselves—at all career stages, but perhaps in particular enabling early career researchers to be heard, given that they are often most acutely aware of the problematic nature of some of the incentives that currently exist in academia.

## Abbreviations

BBSRC: Biotechnology and Biological Sciences Research Council
MRC: Medical Research Council
UKRN: UK Reproducibility Network
